# Data on energy consumption and Nearly zero energy buildings (NZEBs) in Europe

**DOI:** 10.1016/j.dib.2018.11.094

**Published:** 2018-11-23

**Authors:** Delia D'Agostino, Livio Mazzarella

**Affiliations:** a European Commission, Joint Research Centre (JRC), Ispra, Italy; b Energy Department, Politecnico di Milano, Via Lambruschini 4, 20156 Milano, Italy

## Abstract

This data article refers to the paper "What is a Nearly zero energy building? Overview, implementation and comparison of definitions" (D׳Agostino and Mazzarella, 2019). Data linked with this article allow an overview of the European status in relation to energy consumption and energy savings in buildings. Further data are available in relation to Nearly zero energy buildings (NZEBs).

Data relate to primary and final energy consumption, detailing electricity, gas, oil, coal, heat, and wood consumption from 1994 to 2014 in different countries. Data also relate to the stock of constructions of total, single-family, and multi-family dwellings in Europe. Energy consumption per different energy carriers is also given for the residential sector in European and non-European countries. Energy consumption and savings trends can be visualized. In relation to NZEBs, selected retrofit case studies in Europe are presented. Data include energy consumption, saving percentages, costs, payback period, implemented efficiency measures and renewables. Further data is available about building geometry, costs, envelope, lighting, appliances and systems.

**Specifications table**

**Table:** 

Subject area	*Engineering*
More specific subject area	*Building stock data, energy consumption, energy performance*
Type of data	*.xlsx*
How data was acquired	*Data collected from different sources*[2], [3], [4], [5], [6], [7], [8], [9], [10], [11], [12], [13]
Data format	*Processed, formatted*
Experimental factors	*Data formatted for consistency across Member States*
Experimental features	*Data collection on building consumption and NZEBs*
Data source location	*European Member States*
Data accessibility	*Data are provided in supplementary materials directly with this article*
Related research article	*Delia D׳Agostino, Livio Mazzarella, What is a Nearly zero energy building? Overview, implementation and comparison of definitions, Journal of Building Engineering, 21 (2019) 200–212,*https://doi.org/10.1016/j.jobe.2018.10.019[1]

**Value of the data**

• The data describe support energy efficiency and policies trends at European level [2] and can be used to have quantitative information on the European building stock in terms of energy consumption, number and type of buildings.
• The data show the implementation of NZEB retrofit across Europe, in particular technological measures for NZEBs in different locations.
• The data give insight on retrofit solutions for envelope, appliances, and systems in European buildings.
• The data can be used to follow energy consumption trends, comparison or further analysis.

## 1 Data

Data on building energy consumption and NZEBs projects are attached to this paper in the form of two excel spreadsheets. The first (named "energy consumption") reports the data related to:

• Number and type of dwellings (total stock, single-family, multi-family) in Europe;
• Energy consumption in the residential sector (electricity, gas, oil, heat, wood, coil, final) both in European and other countries (Australia, Canada, South Korea, United States, Japan, New Zealand);
• Overall primary energy consumption and savings throughout Europe.

The other spreadsheet (named "NZEB retrofit") reports data related to NZEB retrofit projects. The following information is available for each building:

• Location.
• Building category.
• Year of construction and refurbishment.
• Area.
• Consumption after renovation and percentage saving.
• Type of envelope.
• Roof, wall and basement U value.
• Windows type and U value.
• Heating, ventilation, cooling technologies.
• Lighting and control system.
• Renewable sources and percentage.
• Investment costs.
• Financial incentive.
• Discounted payback period.
• NZEB energy performance.
• Reference value or standards eventually established in the country.
• Project/Source.

## 2 Experimental design, materials and methods

Buildings are of strategic importance of European policies aimed at limiting resource depletion and environmental pollution. Although European policies encouraged the construction sector to move towards NZEBs, the majority of NZEBs are still demonstration projects, indicating that a full implementation of the concept is not yet reached [9], [10].

Reported data relate to building energy consumption in European Member States as collected from different sources [2], [3], [4], [5], [6], [7], [8].

Data give an overview of the European status on primary energy consumption and energy savings in buildings, as exhaustively reported in Refs. [11], [12], [13]. Information on climate, such as heating degree days and cooling degree days, has been already given in previous work [14]. Data are detailed per energy carrier. Data on the stock of dwellings are also available. Data are given for the following countries: Austria, Belgium, Croatia, Cyprus, Czech Republic, Denmark Finland, France, Germany, Greece, Ireland, Italy, Latvia, Lithuania, Netherlands, Poland, Portugal, Romania, Slovakia, Slovenia, Spain, and Sweden. For the other European countries data are not available. The available period ranges from 1990 to 2014.

This paper presents some examples of refurbished NZEBs that combine efficiency measures and renewable production [14], [15]. The linked spreadsheet contains information on NZEBs throughout Europe. The following information has been selected to better describe examples of refurbished NZEBs. General information includes: name of the building, country, year of construction, year of refurbishment, area in m^2^ (heated area or net surface), and building category. The different types of buildings are listed below as categorized in Annex 1 of Ref. [16]:

• single-family houses of different types;
• apartment blocks;
• offices;
• educational buildings;
• hospitals;
• hotels and restaurants;
• sports facilities;
• wholesale and retail trade services buildings;
• other types of energy-consuming buildings

The absolute value of energy consumption after the renovation works is also given [17]. The achieved savings are analysed in relative terms (%).

The technical measures have been collected into main areas, such as envelope, heating, cooling, ventilation, lighting system, control system, renewable sources. Other components are basement, roof, external walls and type of windows. Transmittance values are also reported for walls, windows, and basement.

The economic parameters taken into account are two: investment costs (EUR o EUR/m^2^) and discounted pay-back time period (y). Notes about the financial incentives have been included [18], [19]. A NZEBs definition, the reference value eventually established, and percentage of energy covered by renewables are also reported.

Provided data allow an overview of NZEBs case studied. Data show how a wide range of technologies are becoming an integral part of buildings and how technology plays a major role in exploiting the massive potential benefits of reducing building energy consumptions.

Using the data linked with this paper, comparison can be made with other countries and years in relation to energy consumption in building. The analysis generates a snapshot of European building stock [1].

The establishment of a harmonized database of benchmark refurbished buildings, collected through an harmonized methodology which compares values although respecting differences among European countries, could be an important step in future research on NZEBs [20].
