# Supplementary material for: Data on energy consumption and Nearly zero energy buildings (NZEBs) in Europe
Source: Data Brief. 2018 Nov 23;21:2470–4. doi: 10.1016/j.dib.2018.11.094 (PMC6288979; doi:10.1016/j.dib.2018.11.094)
Supplement: Supplementary file 1 — Supplementary material [file mmc1.pdf]

Conflict of Interest and Authorship Conformation Form

Please check the following as appropriate:

- [x] All authors have participated in (a) conception and design, or analysis and interpretation of the data; (b) drafting the article or revising it critically for important intellectual content; and (c) approval of the final version.

- [x] This manuscript has not been submitted to, nor is under review at, another journal or other publishing venue.

- [x] The authors have no affiliation with any organization with a direct or indirect financial interest in the subject matter discussed in the manuscript

- [ ] The following authors have affiliations with organizations with direct or indirect financial interest in the subject matter discussed in the manuscript:

Author's name	Affiliation
DELIA D'AGOSTINO	JRC - European Commission
LIVIO MAZZARELLA	POLITECNICO DI MILANO
